# Is neurodiversity a Global Northern White paradigm?

**DOI:** 10.1177/13623613241280835

**Published:** 2024-09-21

**Authors:** Vishnu KK Nair, Warda Farah, Mildred Boveda

**Affiliations:** 1 University of Reading, UK; 2 University of Greenwich, UK; 3 Penn State University, USA

**Keywords:** decolonial, Global North, Global South, intersectionality, neurodiversity, race, racialisation

## Abstract

In this article, we engage in a critical conversation with scholars of neurodiversity. We emphasize the transformative role neurodiversity has in creating a crucial space for scholarship to emerge within the academy centering autistic voices. Despite this advancement, research addressing neurodiversity has overlooked and failed to engage with important issues of geography (Global South) and intersectionality (racialized neurodivergent other in the Global North). The first issue of geography relates to the marginalization of Global Southern epistemologies in the neurodiversity scholarship. We ask, why has neurodiversity failed to acknowledge Indigenous and Southern epistemologies and consider the evolution of relatively new Northern scholarship as the epicenter of knowledge production? Second, we highlight how intersectional experiences of the racialized other within the Global North are underrepresented and excluded from the neurodiversity scholarship. Homogenization of neurodiversity as “White Neurodiversity Movement” destabilizes the social justice and emancipatory goals of the movement. In highlighting these issues, we call attention toward knowledge systems that exist within the Global South, marginalization of scholarship and voices within neurodiversity scholarship and accentuate the need for this academic community to commit to a serious scholarship rooted and the intersectional experiences of racialized neurodivergent individuals.

**Lay Abstract:**

Scholarship addressing neurodiversity has made enormous progress in challenging and providing alternative narratives to the dominant frameworks of medical model. Although this is a necessary and important development, scholars need to think and act beyond the immediate local context of theory generation (Global North—mainly the United Kingdom and the United States) and examine its impact on the racialized neurodivergent individuals of the Global Majority. This article will provide a decolonial framework that has been missing in the neurodiversity scholarship. The arguments presented in the article aligns well with the goals of critical autism studies and will further inform the knowledge in this area. Through a decolonial lens, this article brings the crucial issue of knowledge production outside of Global Northern countries, specifically, knowledge systems from the Global South that have parallels with neurodiversity. The article frames neurodiversity as part of an interconnected knowledge continuum rather than considering Global North alone as the only loci of knowledge production. Furthermore, it highlights the lack of focus on the intersections between racialisation and neurodivergence and the implications of this for the racialized neurodivergent individuals of the global majority. The article provides new avenues for theoretical discourses to emerge within the academy. It will have important research implications in relation to how neurodiversity will be viewed and framed outside Global Northern countries. The article highlights the importance of engaging in intersectional and interdisciplinary research and establishing a critical link with the scholars of neurodiversity, critical autism studies, and disability critical race studies.

The title of this article may unsettle some readers as it appears to question the conceptual legitimacy of neurodiversity. We thus begin by clarifying our position in relation to attempts to discredit the epistemological validity of neurodiversity and the social justice movement that emerged out of it (e.g. Bolton, 2018; Guest, 2020). First, we are three scholars institutionally situated in the Global North, with familial and embodied ties to the Global South. Second, Authors 1 and 2 identify as neurodivergent. Moreover, our espoused ideologies and the arguments presented in this article are firmly rooted in the contemporary neurodiversity scholarship (e.g. Bottema-Beutel et al., 2021; Chapman & Carel, 2022; Fletcher-Watson, 2022; Milton, 2020; Pellicano & den Houting, 2022). We reject pathologization of neurodivergent individuals based on the arbitrary binaries of “typical and atypical” or standard norms rooted in Galtonian ideologies. Galtonian ideologies view ability as biologically determined and White Western European heteronormative neurotypical men as superior to racialized and neurodivergent individuals (e.g. Chapman, 2023; Nair et al., 2023). Although creation of such standardized norms has had a disproportionate impact on neurodivergent individuals in the Global North, we highlight that such ideologies have been already used to colonize, subjugate, and produce disablement of much of the population in the Global South (Grech, 2015). Therefore, our critique of neurodiversity is specifically aimed at the dominant theoretical frameworks, their lack of focus on the Global South and the epistemic erasure and inferiorization of non-Northern knowledges (Meekosha, 2011). Furthermore, we highlight the marginalization of experiences of colonized “other” (Black, Brown, and Indigenous individuals) living in the Global North in the current neurodiversity scholarship. We therefore call for a more nuanced understanding of neurodiversity that is situated at the intersection of racism, ableism, White supremacy, colonialism, imperialism, patriarchy, capitalism, and other marginalizing forces.

## Neurodiversity as a Global Northern paradigm

It has been argued that autism research is dominated by reductive psychological and cognitive theories that dehumanize autistic individuals as subjects lacking feelings and empathy among other things (e.g. Botha, 2021; Milton, 2012). Scholars of Critical Autism Studies (CAS) indicate the importance of shifting research from a positivist psychological, cognitivist, and biomedical lens to one that examines how power influences knowledge production (e.g. O’Dell et al., 2016; Woods et al., 2018). CAS as a field is concerned with diverse perspectives in its interrogation of power ranging from challenging cognitive normalcy, examining the construction of autism across different cultures, dismantling the hegemony of non-autistic researchers, and centering the scholarship of autistic autism researchers (e.g. Davidson & Orsini, 2013; O’Dell et al., 2016; Woods et al., 2018). Through the lens of CAS, O’Dell et al. (2016) have problematized the domination of Anglosphere and other Western European countries in the knowledge production relating to autism.

Although this is a noteworthy step, CAS and the wider neurodiversity literature insufficiently regards the impact of colonialism on Global Southern knowledge system(s). A decolonial lens is crucial when considering the current theoretical constructs of autism and neurodiversity as they have been exclusively viewed as emerging from the Global North. While decolonial scholars have extensively written about the current structures of colonialism (i.e. how coloniality have been integrated and applied into the structures of scholarly knowledge production; Quijano, 2000), this debate, in relation to neurodiversity or CAS is non-existent and non-published in English language journals. Scholars have argued that the locus of knowledge production is exclusively designated to the North and the knowledge is expected to flow to the South because the former claims to hold more valid, rational, and universalizing status (e.g. Meekosha, 2011; Menon, 2015). The South is positioned as the passive other capable of knowledge absorption but not production (Menon, 2015). It is precisely because of this history, and the continuous domination of North over the South through coloniality, that critical scholars have called disability studies “Euro-centric disability studies” and cautioned against bypassing colonialism in disability scholarship (Grech, 2015; Grech & Soldatic, 2015).

We extend similar criticisms of disability studies to neurodiversity scholarship (Grech, 2015; Meekosha, 2011). We offer this critique within the theoretical framework of CAS in examining how colonial ideologies that were imposed onto the Global South and to the Indigenous and enslaved people in settler colonial contexts (e.g. Australia, USA, and Canada) have erased plurality of knowledge systems including those who may have explained disability radically different than that of the medical model or a pathology paradigm. For instance, Walker (n.d.-a) argued that a neurodiversity paradigm is premised on the foundational principles of diversity as natural and normative ideologies of cognitive functioning as culturally constructed. We agree with Walker on these principles—however, if one transverses back in time and explores epistemologies of colonized subjectivities, rich philosophical traditions that already align with these ideas can be located. For example, Sefotho (2021) argued that Basotho ontology of disability based on the philosophy of Ubuntu have been subjugated for centuries by the Euro-Western hegemony. Basotho ontology, although acknowledging the stigma and cultural struggles associated with disability, still upholds a principle of diversity and equity where child-rearing, specifically, caring for neurodivergent children is based on community and relational ethics that includes specific care duties assigned to siblings.

Lovern (2022) indicated that relational ethics of Indigenous epistemology does not categorize people into “normal/abnormal” or “good or bad” binaries. It is based on a principle of interdependence where bodymind differences exist because such diversity is at the core of learning and teaching. Differences exist as they allow for gaining wisdom through evolution of plurality of knowledge(s) (Lovern, 2017). In many Indigenous and Southern communities, the bounding feature of unity does not judge a person for what they lack but how they use their skills for maintaining the reciprocal relationship within the community (Ineese-Nash, 2020; Lovern, 2017). Unity refers to the relationship one has with the community where instead of viewing neurodivergence as individuals needing to go through an individualized “remediation” plan, the elders, traditional healers, and families come together and engage with the physical and spiritual world for receiving scared wisdom about the unique embodied experience (e.g. Ineese-Nash, 2020). For centuries, these practices have been denigrated by colonizers and many knowledge systems have been made to disappear due to the assumed superiority of Euro-American epistemology. Indigenous and Southern epistemologies that have survived colonialism offer different views on autism than individualized medical model (see Kapp, 2013 for Navajo philosophy on autism and Hickey & Wilson, 2017 from a Mäori perspective on disability).

The neurodiversity theory and movement have been argued to have started in the 1990’s with autistic individuals organizing using cyber space and with the scholarship of Judy Singer (Chapman, 2023). Recently, scholars have highlighted the importance of acknowledging the diverse, complex, and collective history of neurodiversity identifying the contribution disability scholars such as Kassiane Asasumasu and Anita Cameron rather than attributing the concept to Singer alone (Botha et al., 2024). However, even within this scholarship, a relatively recent temporal conceptualization from Global North is outlined that has overlooked Southern epistemologies or other knowledge systems that have existed even within the Global North (e.g. Sami knowledge systems, Maori philosophies). We argue that a new temporal and scholarly conceptualization of neurodiversity should emerge that acknowledges Southern and Indigenous knowledge systems preceding and having parallels with the current Northern scholarship. While there are enormous power differences between these knowledge systems, a decolonial lens challenges us to integrate them as part of an interconnected knowledge continuum beyond conceptualizations of the North as the beginning and only loci of knowledge production.

It is important to highlight that our intention is not to homogenize or essentialize Southern or Indigenous epistemologies or neither do we make the argument that they cannot be critically examined because these communities had radically different understanding of disability. However, we underscore how these diverse epistemologies have predated the current Northern neurodiversity paradigm. They not only have parallels with it but also go deeper in its holistic understanding of disability, non-reductionism, diversity, community, relational ethics, and spiritual and physical well-being. We do not seek to undermine the foundational work of scholars and activists on neurodiversity in the Global North. However, if social justice is at the core of neurodiversity movement, then it must be grounded in its understanding that current scholarship of neurodiversity is parochial in its scope in that they are derived primarily from White scholars in the Global Northern institutions. Advancement in the discourses of the North was made possible only through the material and intellectual resources from the South (Gopal, 2021). Northern scholars cannot overlook the fact that there is an ongoing epistemological marginalization and epistemicide^1^ of the Southern and Indigenous worldview that have directly contributed to the Northern dominance in scholarship.

The neurodiversity paradigm and movement have an ethical, moral, and scholarly responsibility in addressing the colonial histories of exclusion, power, and contemporary marginalization of “othered” knowledge(s) within the Northern academy. Although there have some recent attempts to support global autism research (see editorial by Cheng et al., 2023; also see Divan et al., 2024), a focus on neurodiversity through a decolonial lens, must be taken up within the CAS as it is commensurate with its foundational goals of examining power (e.g. Milton, 2014; O’Dell et al., 2016). Examining how cultural construction of neurodivergence vary across spatial locations and documenting how geographies viewed neurodivergence prior to colonization will enrich and expand the current scholarship on autism and may unsettle the notion of neurodiversity as exclusively Northern.

We must caution that understanding neurodiversity from Southern contexts does not indicate assimilation of these diverse epistemologies into the Northern scholarship. Decolonial disability scholars have critiqued importing disability studies or social model of disability to non-western contexts (Ghai, 2002; Grech & Soldatic, 2015). The current paradigm(s) of neurodiversity emerging out of Northern institutional spaces cannot be imposed on the Global South to extract Southern knowledges and integrate them into Northern frameworks. Instead, the recognition that (sense-making about) disability existed and continues to exist in all societies is critical. Thus, a global perspective would go beyond a singular notion of neurodiversity, acknowledging the plurality of how societies have imagined diversity of body minds (Canagarajah, 2023). Instead of adopting a liberal neurodiversity paradigm to post-colonial societies, one must interrogate the relevance of current neurodiversity paradigms to Southern communities. For example, Canagarajah (2023) examined applied linguistics and argued that scholarship from the North focuses on the individual competence and communication outcomes of disabled individuals whereas South values distributional practice emphasizing ethical and moral responsibility toward understanding and caring for neurodivergent individuals. This does not imply there is no marginalization of neurodivergent individuals in the South—however, the knowledge of distributional practice within Southern communities and their action arising out of relationality and community-based values cannot be negated (see Canagarajah, 2023 for more on distributional practice).

We argue for the need to move toward serious scholarship about the diverse distributional practices existing within the Global South and away from universalizing and uncritically adopting Northern neurodiversity paradigm(s) to all contexts. This commitment entails understanding local contexts, particularly, how intersectional social markers (e.g.,, caste, religion, gender or socio-economic status), shape practices of the Southern communities and are entangled with the social mechanisms threatening community care for disabled individuals in Southern contexts (see Grech, 2011 discussion on global capitalism).

## People of color: invisible or invisibilised? (White) neurodiversity movement

We have argued that Global Northern hegemony in neurodiversity scholarship has marginalized other global epistemologies. We extend this criticism to the marginalization of intersectional experience of race and neurodivergence, that is, neurodivergent racialized bodies within the Global North (e.g. large proportion of Black and Brown individuals who are descendants from colonized countries in the Global South and Indigenous individuals). For instance, within the literature addressing neurodiversity, very few research has highlighted intersectionality as a crucial point of analysis in understanding autistic experience. This indicates a serious intersectional analysis is neglected in the neurodiversity scholarship (e.g. Botha & Gillespie-Lynch, 2022; Brown, 2017; Brown et al., 2017; Onaiwu, 2020). A recent review examining intersectionality within CAS has found less than five articles focusing on neurodivergence in racially minoritized communities (Mallipeddi & VanDaalen, 2022).

The marginalization of intersectional experiences of racialized neurodivergent individuals is disconcerting, given that foundation of neurodiversity is built with a recognition of intersecting identities. For instance, Strand (2017) argued that the foundational ideologies of neurodiversity acknowledged that it is akin to other forms of diversity intersecting with identities such as gender, ethnic, and cultural diversity. While the concept has been used in building neurodiversity theory, Strand (2017) suggests that an explicit reference of intersectionality is missing from its originating discourses. This is especially crucial given Black feminist thinking has been fundamental to the conceptualization of intersectionality, specifically, in examining how intersecting identities of race and gender contributes to the oppression of Black women in the US jurisprudence (Crenshaw, 2013). Similarly, Black feminist thinking and ideas have been applied for rejecting the pathology paradigm and envisioning liberation for autistic individuals. For instance, Walker (2012) utilized celebrated Black writer and philosopher Audre Lorde’s critique of racist patriarchy in exposing the systemic oppression of the pathology paradigm and charting a way ahead for autistic empowerment. Lorde (1984) critiqued White feminism for its exclusion of racism, queerness, and the experience of Global Southern women from its analysis. Lorde (1984) argued that the liberation envisioned by White feminism is rooted in racist patriarchy undermining the intersectional experiences of the most marginalized women of color which can only bring minimal change that will not dismantle master’s house.

Inspired by Lorde (1984), Walker (n.d.-b) argued that

Genuine, lasting, widespread empowerment for Autistics can only be attained through making and propagating the shift from the pathology paradigm to the neurodiversity paradigm. We must throw away the master’s tools.

We argue that to throw away the master’s tools utilizing a neurodiversity paradigm, one must first critique the dominant discourse on neurodiversity and its own complicity in racism. We apply Lorde (1984) criticism and ask, where are the Black, Brown, Indigenous, and other marginalized scholars in this movement? It is ironic that a movement that owes its foundational conceptualization to Black feminist thinking has not acknowledged its evasion of race, racism, and intersectional experiences of neurodivergent individuals of color. We recognize that a special focus has been paid to examining the intersections of neurodivergence and gender, specially concerning the barriers of autistic women in relation to social inclusion and receiving a diagnosis (e.g. Saxe, 2017). This is a necessary analysis, however, there can be no serious analysis of neurodivergence and gender without accounting for the experiences of Black, Brown, and Indigenous individuals.

A lack of commitment toward examining the intersections of racialization and neurodivergence is indicative of the overrepresentation of Whiteness in the neurodiversity scholarship. The entanglement of race and neurodivergence and the exclusion of these intersections are a serious omission both from a neurodiversity scholarship and within the growing body of literature on CAS. We believe this omission is not accidental. Similar to what Lorde (1984) argued almost 40 years ago, without a serious intersectional analysis of race and gender, the neurodiversity movement will be depoliticized and weakened in its goal toward autistic empowerment as envisioned by Walker (n.d.-a).

Although we have pointed out the dominance of Whiteness in scholarship, we do not reduce the lack of focus on intersectionality to be a problem only in relation to academic knowledge production. As scholars who live at the intersections of racialization, neurodivergence, queerness, and other multiple marginalized social identities, we have firsthand experienced marginalization and oppression within higher-education spaces. For instance, the first two authors are trained speech and language therapy clinicians, a profession recognized as the fourth Whitest profession in the United States (Yu et al., 2022) and has the second-highest White registrants in the UK health care (Nkomo et al., 2022). The White academy has always been hostile to our existence, for instance, the first author with his intersecting identities of racialization, neurodivergence, and queerness was asked to stop discussing race when advocating for racialized neurodivergent multilingual children and families because it made a few White colleagues feel uncomfortable. The second author was targeted and characterized as “dangerous” for her brave disposition of owning her own identity as a racialized neurodivergent woman and using that experience to expose anti-blackness in speech and language therapy (Brea-Spahn et al., 2022). As racialized neurodivergent scholars, we have felt the negative consequences of our own intersectional lived experiences to be afforded a space controlled and regulated by the existence of Whiteness.

In a special issue addressing Black Lives Matter and Education Industrial Complex, Aronson and Boveda (2017) raises the question of “do Black lives matter in the US education industrial complex?” We rephrase this question and ask “do Black and Brown neurodivergent Lives Matter in European metropoles and settler colonials States?” Here, we point to the final and crucial issue of police violence on neurodivergent Black and Brown bodies. Intersectionality provides a crucial theoretical framework that reveals how the violence of policing and legal systems on racialized bodies can never be explained through single categories of race or neurodivergence alone—but only through carefully analyzing the impact of the “convergence of the whole” on the *othered* (Collins, 1998; Crenshaw, 2013). We refer to the important and extensive work in this area by critical disability scholars who have indicated the blurred boundaries between race and ability (Annamma et al., 2013; Artiles et al., 2002; Erevelles & Minear, 2010). The violence on Black autistic bodies such as deportation of Osime Brown (Bulman, 2020) or the incarceration of Matthew Rushin and Emmanuel (Oyeri, 2021; Rozsa, 2020; also see Vance, 2019) strongly suggests that the boundary between neurodivergence and race is equally reduced. To fully understand this requires neurodiversity and CAS scholars to establish a critical link with intersectional scholarship on disability critical race studies (Annamma et al., 2013, 2018; Boveda et al., 2023; Coard, 1971; Erevelles, 2014).

There are conceptual similarities between critical disability scholarship and the concepts of neurodiversity. Critical disability scholars offer tools for understanding the intersections between race and disability and have critiqued the notion of disability, particularly categories of language and cognitive disabilities, as biological or medical problems (e.g. Annamma et al., 2013). These scholars reject ideologies of pathologies and abnormalities rooted in individual body-minds and argue for the examinations of disability as a political and social category. These arguments align with the foundational notions of neurodiversity, which reject the biomedical model or pathology paradigm. Disability critical race scholars, however, offer a deeper understanding in exposing the ableist ideologies rooted in the construction of race. They view disability and race as mutually reinforcing rather than exclusive ideological categories. We argue that a focus on intersectionality examining race and neurodivergence and other intersectional markers integrating critical disability scholarship will strengthen the theoretical understanding of neurodiversity, as well as advance the social justice goals envisioned within neurodiversity and CAS. Failing this would conceal the structural factors that create hierarchy in devaluing Black and Brown neurodivergent lives compared to White neurodivergent lives—leading to *Neurodiversity reduced to White Neurodiversity.*

## Conclusion

We problematize the status of neurodiversity as exclusively Northern knowledge and how its universalizing status and temporal conceptualization contributes to the marginalization of Southern and Indigenous epistemologies. We underscored the current scholarship on neurodiversity has an overrepresentation of Whiteness that erases the intersectional experiences of Black and Brown individuals. While our perspective offers a critique of current neurodiversity scholarship, our aim is to also provide a decolonial perspective to the current conceptualization of neurodiversity. Decolonization is needed for both Northern academic communities as well as for Southern scholars who apply Northern paradigms uncritically in post-colonial contexts.

The first step toward challenging this trend is to unmask neurodiversity as a Global Northern parochial conceptualization particularly emerging from countries such as Australia, USA or the UK. This theoretical critique will recognize the need to rediscover and understand other global epistemologies that challenge the hegemonic notion of knowledge production attributed to Global Northern countries. Doing so liberates and reconceptualizes neurodiversity as *one of* the theories explaining diversity of bodyminds and as *part of* a vast global knowledge continuum rather than a singular dominant Western notion imposing itself onto other knowledge systems (Canagarajah, 2023; Shiva, 1993 also see Henner & Robinson, 2023 for other Northern frameworks such as crip linguistics). In our final critique, we have highlighted that neurodiversity in the Global North needs to urgently focus its attention toward the pathologization and dehumanization of racialized individuals. This omission is a crucial deterrent as we have illustrated how material structures are inexplicably oppressive and violent toward racialized neurodivergent individuals. We call for scholars of neurodiversity and CAS to cross disciplinary boundaries in understanding the intersections between race, racialization, and neurodivergence among other social markers of difference.

## Notes

1. Epistemicide refers to the destruction and killing of othered and colonized knowledges (Hall & Tandon, 2017). See Grosfoguel (2013) for a detail of theoretical construct on this including a description of four different types of epistemicide.
